# 

**Published:** 1936

**Authors:** 


### CONTENTS.

*Page*

The Twenty-fifth Long Fox Memorial Lecture: Somerset Lake Villages. By Dr. A. Bulleid, F.S.A. .. .. .. 187

Abdominal Pain in Childhood. By Wilfrid Sheldon, M.D., F.R.C.P. .. .. .. .. .. .. .. .. .. 219

The Treatment of Tobacco Amblyopia by Acetyl-Choline. By B. H. Cragg, L.M.S., M.D. .. .. .. .. .. .. 237

The History of the Bristol Medical Dramatic Club. By A. L. Flemming, M.B., Ch.B .. .. .. .. .. .. .. 239

Reviews of Books .. .. .. .. .. .. .. .. .. 247

Editorial Notes .. .. .. .. .. .. .. .. .. 257

Meetings of Societies .. .. .. .. .. .. .. .. 260

The Medical Library of the University of Bristol .. .. 263

Publications Received .. .. .. .. .. .. .. .. 264

Local Medical Notes .. .. .. .. .. .. .. .. 265

